# Children’s Digital Art Ability Training System Based on AI-Assisted Learning: A Case Study of Drawing Color Perception

**DOI:** 10.3389/fpsyg.2022.823078

**Published:** 2022-03-21

**Authors:** Shih-Yeh Chen, Pei-Hsuan Lin, Wei-Che Chien

**Affiliations:** ^1^Department of Computer Science and Information Engineering, National Taitung University, Taitung, Taiwan; ^2^Department of Management Information Systems, National Chung Hsing University, Taichung, Taiwan; ^3^Department of Computer Science and Information Engineering, National Dong Hwa University, Shoufeng, Taiwan

**Keywords:** AI-assisted learning, augmented reality, STEAM education, technology art, drawing assessment

## Abstract

This study proposed a children’s digital art ability training system with artificial intelligence-assisted learning (AI-assisted learning), which was designed to achieve the goal of improving children’s drawing ability. AI technology was introduced for outline recognition, hue color matching, and color ratio calculation to machine train students’ cognition of chromatics, and smart glasses were used to view actual augmented reality paintings to enhance the effectiveness of improving elementary school students’ imagination and painting performance through the diversified stimulation of colors. This study adopted the quasi-experimental research method and designs the pre-test and post-test for different groups. The research subjects are the Grade 4 students of an elementary school in Taitung City, Taiwan. The test tools included an imagination test and an evaluation of painting performance ability. The test results of a total of 30 students before and after the experiment included the experimental group that received the children’s digital art ability training system with AI-assisted learning and 30 students in the control group that had not received the teaching were analyzed by covariance. These results were supplemented by the description and interpretation of student feedback, teachers’ reflection notes, and other qualitative data to understand the performance of the students in the experimental group in terms of imagination and painting performance.

## Introduction

The 21st century is the century of esthetics, and under the trend in this general environment, the United Nations Education, Scientific and Cultural Organization (UNESCO) launched a mid- to long-term esthetic education program. With the concept of esthetics being life itself, by cultivating esthetics from an early age, conducting cross-domain integration and international connections, and by connecting art with campus and daily life, it is hoped that students can be trained to experience beauty through the process of discovery, exploration, and experience starting from the curriculum, activities, and learning environment. This program also seeks to improve children’s esthetic quality and apply esthetics to their daily lives for a better life (UNESCO, 2010). Art education is a component of basic, sustainable, high-quality, and updated education. Especially as a part of extensive and comprehensive education, the importance of art can be learned from sustainable education through international organizations. As the future of education moves toward 2030, the realization of inclusive and fair quality education for all should be ensured, and lifelong learning should be promoted to achieve the educational goal of sustainable development (United Nations Educational, Scientific, Cultural Organization, 2016). New ideas are frequently presented in the innovations and inventions in science and technology in Taiwan. Through the combination of esthetic education and digital technology, more forward-looking cross-disciplinary talents can be cultivated to add value to Taiwan’s industries. Therefore, the combination of esthetic literacy and technological capabilities has become a source of national competitiveness and creativity, which is in agreement with the concept of “The Future of Education and Skills: OECD Education Framework 2030,” as published by the Organization for Economic Co-operation and Development, OECD (2016). Civil society leaders and policy leaders in UNESCO recognize and provide art education resources as an indispensable pillar for promoting and cultivating a culture of sustainable social development (Education, 2019). Painting is one of the most common ways of practicing art, and its expression can be traced back to ancient times. Paintings help us understand how the world thinks and feels, its shapes, and how it communicates, they are the simplest and most effective way to convey visual thoughts. Paintings are interesting, easy to understand, and priceless (Makowska, 2012; Quillin and Thomas, 2015).

Painting is a universal language, and can usually be understood wherever it goes (Gilbert, 1998). It is the first way of expression and presentation invented by mankind, and it is still very effective whether as an artistic expression of reality or as a way of expressing pure imagination. People must be able to see, understand rationally, and feel emotions to master the skills that enable us to fully express our thoughts and emotions (Caranfa, 2001; Robinson, 2004). The development of technology has changed our mode of living and thinking. Technology brings convenience to human life and allows us to enjoy life while cultivating our temperament and creativity in esthetics and art activities. Therefore, painting constitutes the foundation of all artistic creation and technological development and is one of the most important research branches in colleges of architecture, engineering, and other sciences. The foundation of visual art is the performance and skills of painting, and perfection is achieved through continuous practice and dedication. Painting is the essence of the expression of artists and designers, and an effective way to communicate and think, thus, painting operates on many levels. Artists and designers must understand these differences and achieve a certain level of skill in the painting discipline (Ernest, 2006). Designers often use a lot of sketches when generating design ideas. In the first stage of the creative design process, it is particularly important to use drawings and sketches to search for alternative design plans, which has a positive effect on expanding the scope of design ideas. Researchers of design thinking regard this kind of sketching activity as a means to stimulate creative thinking, and interactions between designers and their sketches are regarded as a necessary condition for creativity in design activities (Van der Lugt, 2002). Drawing allows designers to simultaneously consider several alternative design concepts (Tovey, 1989). While the development of science and technology was originally for the benefit of human life, when technology developed to a certain extent, we began to consider the nature of human beings. The nature of human beings cannot be separated from life, life cannot be separated from culture, and culture cannot be separated from art, thus, the combination of technology and art has become an inevitable trend. Like other space construction tasks, such as module block assembly, drawings can help us to deeply understand how individuals copy each part of an array, as well as the relationship between each part, in order to construct the whole configuration (Stiles et al., 2013), thus, it is considered to be a generative learning activity that promotes student participation (Ligorio et al., 2016). Figurative drawings have long been adopted in the research field of artificial intelligence as a common related object of research and analysis from which to obtain meaningful results (Minsky and Papert, 1972). Thus, by combining Picasso’s cubism and the neoclassical style to depict images of women, this study proposed and implemented a children’s digital art ability training system based on AI-assisted learning, used smart glasses as a guided learning tool to teach drawing and color recognition, and explored the following issues:

1. Can the children’s digital art ability training system with AI-assisted learning enhance the imagination of elementary school students?
2. Can the children’s digital art ability training system with AI-assisted learning improve the painting performance ability of elementary school students?

## Literature Review

### Science and Art in Science, Technology, Engineering, Art, and Mathematics Education

Science, Technology, Engineering, Art, and Mathematics (STEAM) is a developing education model that organizes traditional academic subjects, science (S), technology (T), engineering (E), art (A), and mathematics (M) into a framework, and a comprehensive curriculum is planned according to this framework (Yakman, 2008). STEAM supporters claim that the framework increases the demand for classroom teaching innovation, and more precisely, it helps break the traditional boundaries between disciplines. The difference between STEAM and STEM lies in that the education strategy of the former is based on adding art to STEM subjects to incorporate various subjects belonging to the humanities, social sciences, and fine arts, which can make mathematics, science, technology, and engineering more attractive and appealing to students, especially those who are traditionally underrepresented in STEM subjects (Peppler and Wohlwend, 2018). The addition of these subjects emphasizes the importance of creativity in student development and learning, and is considered essential for progress and innovation (Perignat and Katz-Buonincontro, 2019). STEAM is characterized by seeking meaningful learning and stimulating students’ convergent thinking (common in STEM subjects) and divergent thinking (common in art) (Yakman and Lee, 2012). Another feature of STEAM is that it allows students to play an active, constructive, and key role in learning and promoting collaborative works (Chien and Chu, 2018; Thuneberg et al., 2018), which aims to encourage students to build personal value, self-efficacy, confidence, and impetus in technological learning (Clapp and Jimenez, 2016), explore the learning environment and connect the knowledge content of multiple disciplines (Quigley et al., 2017). STEAM has positive impacts on improving students’ interdisciplinary ability, professional interest, and learning attitude, and innovative teaching practices at different levels of education have been developed (Khine and Areepattamannil, 2019). The combination of art activities or products is intended to introduce “fun” in science classes, which can be an approach to clarify scientific concepts and show its relevance to students’ daily lives (Ozkan and Topsakal, 2020). At the same time, STEAM can improve students’ attitudes toward science (Kim et al., 2014), while simultaneously promoting creativity, innovation, critical thinking, empathy, and effective communication (Catterall, 2017; Allina, 2018).

Some studies have pointed out that the low level of preparation by teachers in designing and providing comprehensive courses are the basic obstacles of STEAM education. More specifically, teachers lack a proper understanding of the concept of curriculum integration (Radloff and Guzey, 2016), as well as the knowledge and abilities required by the different subjects of the acronyms that constituent STEAM (Shin and Han, 2011; Toma and Greca, 2018). Teachers also have difficulties in selecting suitable topics, developing educational materials, and/or assessing students’ learning results (Hong, 2016). Coupled with the previous development policy of STEM education, which has led to the declining quality of art education in most parts of the world, this lack of teacher ability has an impact on students’ learning, as it fails to give them humanity literacy or a complete worldview. In recent years, the academic world has begun to show interest in encouraging the close connection between the humanities and science and technology, and considers it one of the keys to human development. This exploration of integration responds to the need to provide a more comprehensive education for new generations, as it requires a mix of scientists and technical experts, as well as professionals in the arts, humanities, and social sciences to capture and understand the nuances and interpretations of human behavior (Hartley, 2017). Integrated art courses can create viable art publicity by providing more relevant and useful positions, which would promote the application of art and design in school curricula. Combining art with other courses, such as mathematics and science, may increase the chances of obtaining arts in education (Arts Education Partnership, 2018). Therefore, in order to improve the quality of arts education, the STEAM education strategy adopted in this study is different from previous research that integrated art into STEM disciplines. Instead, as learning appropriate painting skills can improve the effectiveness of many technical design fields, STEM disciplines were applied to learn painting in art education. STEAM is an auxiliary skill of the visual communication form that is considered indispensable for engineering professionals (Danilova and Pudlowski, 2009), and can improve people’s thinking about structures, shapes, and surrounding environments, which has great advantages in terms of providing better design results (Białkiewicz, 2019). At the same time, artistic practices have socio-emotional components, including self-discipline and collaboration, which will significantly affect the performance of K-12 students (Workman, 2017).

This AR based digital art ability training system with AI-assisted learning allows children to play in the digital world, while connected to the real world, and contains science, technology, engineering, art, and mathematics (STEAM) education. Yakman (2010) indicated that the STEAM framework, which combines science, technology, engineering, art, and mathematics with interdisciplinary teaching methods, allows students to engage in hands-on work to construct projects, and present the art according to the foundations of mathematics and science. In this study, the dimension of science is proportion of mixing pigments, the dimension of technology uses AR and AI technology, the dimension of engineering allows students to sequence and draw the interactive learning environment, the dimension of art allows students to arbitrarily paint and create the learning environment.

### Application of Augmented Reality to Innovative Learning

Augmented Reality (AR) is a kind of computer modeling and simulation technology that organically integrates the real world and the virtual world, which enables people to interact with machine vision or other sensory environments, and brings a better sense of reality and improved interactivity (Madden, 2011). AR is a 3D technology that enhances the user’s perception of reality by generating a contextual information layer, thereby improving the user’s sensory perception in the real world (Cuendet et al., 2013), as well as a technology that superimposes virtual objects in the real world, in order that people can feel the real coexistence of virtual objects in the surrounding reality (Akçayir and Akçayir, 2017). In most AR applications, users mainly use smart glasses (Başoğlu et al., 2017), smartphones, tablet computers, and other mobile devices to receive virtual images and models (Arnaldi et al., 2018). This technology has been used in many fields, such as games (Aukstakalnis, 2017), exhibitions (Schmidt et al., 2017), industrial assembly (Doshi et al., 2017), healthcare (McCarthy and Uppot, 2019), manufacturing (Egger and Masood, 2020), maintenance (Siew et al., 2019), and learning programming and computational thinking (Lin and Chen, 2020), while the field with the most potential for development is the field of education (VanDerSchaaf et al., 2021). This technology provides learners with excellent teaching opportunities, including mobility, visualization, alternative perspectives, multi-perspective comparison, contrast, and integration. Through related formative evaluation mechanisms, AR-based learning significantly improves learners’ achievements and motivation, while reducing their cognitive burden (Muliyati et al., 2019). Therefore, AR is regarded as one of the most promising technologies to support K-12 education and higher education (The New Media Consortium, 2017). At the same time, the latest research and development of AR technology can enable its combination with other technical concepts, such as machine learning, artificial intelligence, and deep learning.

## Research Method

**FIGURE 1:**
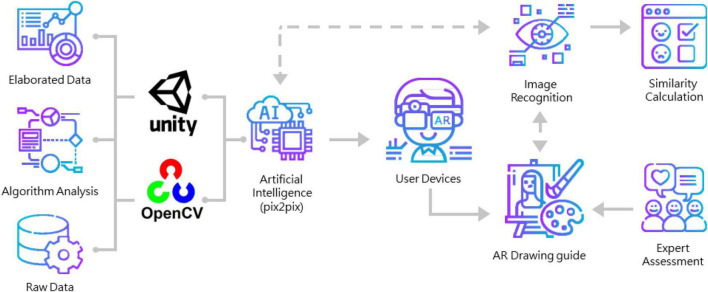
System architecture diagram of children’s digital art ability training system based on AI-assisted learning.

In order to improve children’s painting ability, a children’s digital art ability training system with AI-assisted learning was proposed by this study. The system combines the architecture of smart glasses and edge computing, and transmits the images seen by the children to the edge computing platform through the smart glasses. The system performs outline recognition with Open CV (Bradski and Kaehler, 2008) and generates the recommended hue color matching with pix2pix (Isola et al., 2017), and finally, it calculates the toning ratio based on the color generated by pix2pix and returns the recommended color matching message to the smart glasses to guide learners to learn color recognition and draw at the same time. The system architecture diagram is shown in Figure 1. The following is an introduction to the AI-assisted coloring method adopted in this study.

Generally, there are two image coloring methods, unguided coloring and guided coloring. The former uses an algorithm to directly color the outline, and the latter involves human intervention in the coloring process, such as providing a picture of the reference style to color the outline according to the color distribution of the picture. The other method is to select a specific color for a designated specific area, and then, draw short lines of the desired colors within the outlined areas to color these areas with the automatic filling method. Although these two methods can simplify the coloring process, they both require the assistance of professionals or similar pictures, which leads to extremely high costs and many limitations. Therefore, the image-to-image translation method was adopted by this study to achieve the automatic coloring.

This study used the pix2pix method, which is based on conditional Generative Adversarial Networks (cGAN), to color the outline. The input image can be regarded as a condition in image-to-image translation, and the mapping between the input image and the output image can be learned. The generation of the output image depends on the input image in this method. In other words, the pix2pix model can learn the color matching skills of expert paintings by inputting some expert paintings, and then, mapping this color matching habit to the specified outline, in order to achieve the purpose of image-to-image translation.

**FIGURE 2:**
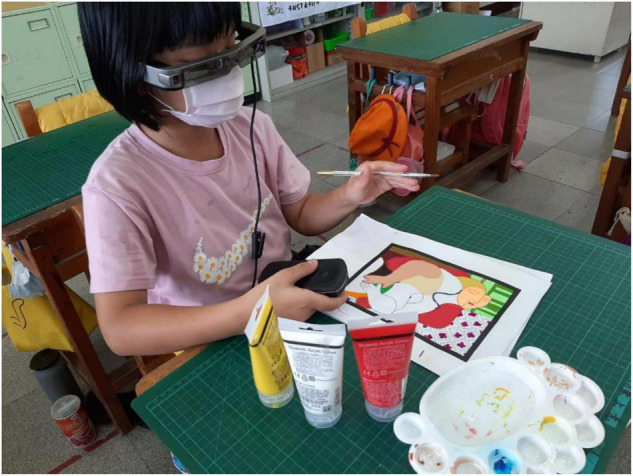
Experimental group learners’ actual paintings through smart glasses.

**FIGURE 3:**
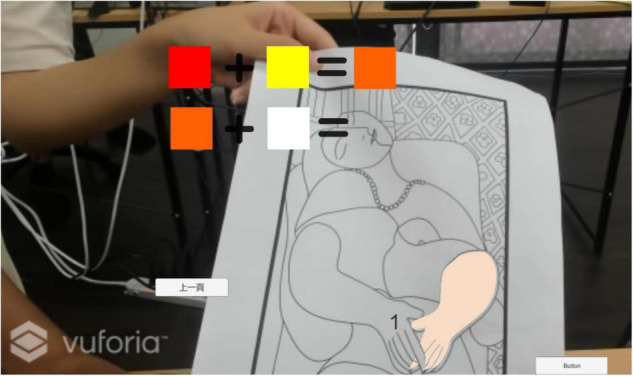
Using AI to assist and guide learners to learn about color recognition and draw at the same time.

**FIGURE 4:**
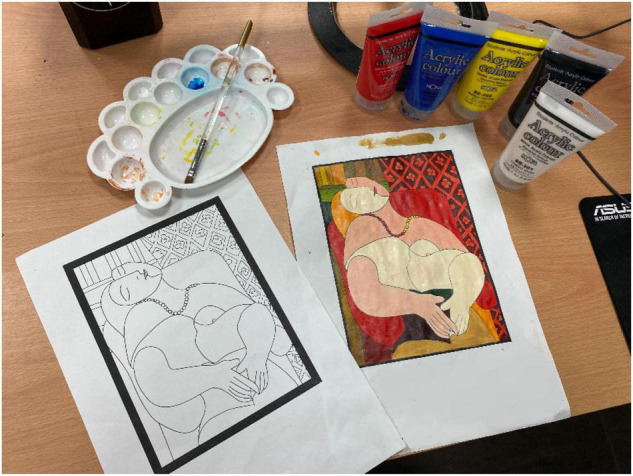
Combining three primary colors (red, blue, yellow) of the pigments with black and white to make up for the shortcomings of the three primary colors.

When the learner puts on the smart glasses, the suggested AR color matching image will not appear automatically. When a learner does not know how to solve the color matching problem and presses the controller button in Figure 2, the system will provide the suggested color matching information and the color ratio (Figure 3), meaning the learner can use the three primary colors of pigments (red, blue, yellow) plus black and white to obtain the system’s recommended color matching (Figure 4). This study used this method to guide and cultivate children’s color matching to allow learners to intuitively associate color matching methods and color matching skills when facing different colors in the future.

## Experimental Analysis and Discussion

**FIGURE 5:**
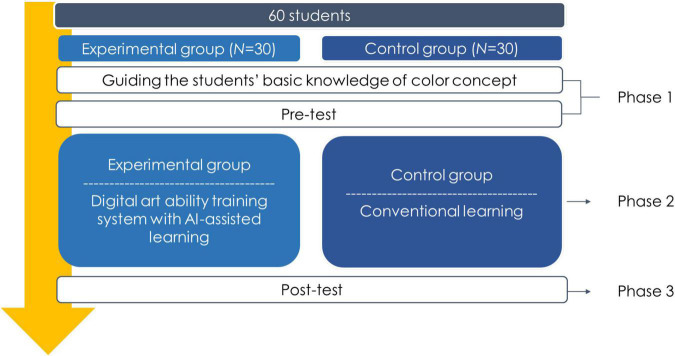
The learning procedure.

This study adopted the quasi-experimental research method and applied the experimental design of pre-tests and post-tests for different groups. The research subjects were the Grade 4 students in an elementary school in Taitung City, Taiwan. The test tools were an imagination test and a painting performance evaluation, and analysis was conducted on 30 students in the experimental group, which received the children’s digital art ability training system with AI-assisted learning, and 30 students in the control group, which did not receive the teaching. Imagination and painting performance were used as the dependent variables in this study. Figure 5 shows the learning activity procedure of the experiment. In Phase 1, the experimental group and control group were both given a course on the basic knowledge of color concept which is the part of regular course learning in art. All the students took a pre-test to evaluate their imagination and painting performance ability. In Phase 2, the students in the experimental group use the digital art ability training system with AI-assisted learning. During the learning activity, the students in the experimental group used the system to draw and finish the picture in actual learning environment, while those in the control group used the pigments and painting tools for the same picture. In Phase 3, after the learning activity, all the students also took a post-test to evaluate their imagination and painting performance ability. The evaluation of subject’s “imagination” was based on their pre-test and post-test scores in the Torrance Tests of Creative Thinking (Torrance et al., 1992); the ability of association and imagination was judged according to the graphics, and the subject’s ability to manipulate mental images when composing new images, inventions or stories was analyzed. The content of the “graphic combination test” is, as follows. The subject was asked to use their imagination to create one new work based on three different three-dimensional geometric figures, the content of creation was not limited, and could include images, invented products, and storylines, and the contents of creation should be named and explained. Cronbach’s α = 0.856, which shows good consistency and reliability.

The evaluation of “painting performance ability” was based on the pre-test and post-test scores of the subjects’ painted works. The evaluation was divided into: (1) similarity was calculated after completion of the painting with the AI-assisted learning system, and (2) evaluation by experts. The criteria were jointly reviewed by teachers with rich teaching experience in the field of visual arts in elementary schools and the professional reviewers of children’s drawing competitions. Based on the opinions of all parties, the final version was completed after revisions and used as the judgment basis for children’s painting performance in this study. Children’s painting performance was scored by three raters that fully understood the evaluation criteria and reached a consensus before determining the total score for each student’s artwork. The evaluation content included three indicators: esthetics and richness, imagination and creativity, childishness and innocence. Cronbach’s α = 0.944, 0.991, and 0.941, which shows good consistency and reliability in the ratings of painting performance. Table 1 shows the summary of Cronbach’s α values.

**TABLE 1:** The Cronbach’s α value of each dimension.

Dimension	Cronbach’s α	Developer
Imagination	0.856	Torrance et al., 1992
Esthetics and richness	0.944	Experts
Imagination	0.991	Experts
Creativity	0.941	Experts

The analysis of one-way covariance results of the two groups of students in the “Torrance Tests of Creative Thinking” can be obtained from Table 2: originality [*F*_(1,58)_ = 10.04, *p* = 0.003], flexibility [*F*_(1,58)_ = 7.38, *p* = 0.011], title abstractness [*F*_(1,58)_ = 4.07, *p* = 0.032], total imagination score [*F*_(1,58)_ = 12.71, *p* = 0.001], the total score of imagination scale [*F*_(1,58)_ = 4.13, *p* = 0.045], and each item reached significant difference. It can be seen that the experimental group, which received the children’s digital art ability training system with AI-assisted learning, all had significantly higher performances in originality, flexibility, title abstractness, and higher total scores than the control group. The research subjects of the experimental group had significant differences in “originality,” “flexibility,” “title abstractness,” and “total score of the scale” in the Torrance Tests of Creative Thinking, which shows that the experimental group, which received the children’s digital art ability training system with AI-assisted learning, scored significantly better than the control group in the above four items of imagination. This children’s digital art ability training system places emphasis on the motivation that arouses students’ imagination, thus, it uses a variety of AR images to accumulate students’ visual experience to guide students to observe and think, and then create, which shows that multiple and fresh visual stimuli can help enhance graphic imagination originality. The imaginative operation mechanism helps to improve the flexibility of graphic imagination. Picasso’s paintings from his Cubist period were used as the exercise content of the system in this study, meaning they are expressed through geometric figures, which coincides with the three dimensions of flexibility scoring in “Torrance Tests of Creative Thinking”: graphics combination/composition, graphics changes, and graphics use, which helped to improve students’ flexibility performance. This finding supports the research result of the experimental group having significantly better flexibility scores than those of the control group.

**TABLE 2:** Summary of the analysis of one-way covariance of Torrance Tests of Creative Thinking for both groups of subjects.

Source	Type I sum of squares	*df*	Mean squares	*F*
Originality	9.35	1	4.17	10.04**
Flexibility	16.33	1	8.95	7.38**
Headings abstract	4.85	1	2.64	4.07*
Total creativity	76.74	1	30.53	12.71***
The Criterion Validity of total creativity	25.48	1	12.78	4.13*

*p < 0.05; **p < 0.01; ***p < 0.001.

The descriptive statistics of the scores of the two groups on their painting performance ability, including the averages of the pre-test and post-test, the standard deviation, and the adjusted average, are shown in Table 3, which shows that the total score of the experimental group in the painting performance ability (*M* = 92.93, SD = 3.27) in the post-test was higher than the total score of the control group in painting performance ability (*M* = 85.58, SD = 3.92). In order to confirm the effect of the manipulation of the experimental variables, the analysis of one-way covariance was performed to exclude the influence of the pre-test variables of the two groups.

**TABLE 3:** Summary of averages, standard deviations, and adjusted averages of both groups in “painting performance ability” in the pre-test and post-test.

	Experiment group (*N* = 30)				Control group (*N* = 30)			

	Pre-test M(SD)	Post-test M(SD)	Adjusted average	95% CI	Pre-test M(SD)	Post-test M(SD)	Adjusted average	95% CI
Total score of drawing performance	85.25 (7.85)	92.93 (3.27)	90.00	[84.97, 93.59]	84.43 (3.62)	85.58 (3.92)	84.18	[85.02, 87.28]

As shown in the analysis of one-way covariance of Table 4, the total scores of the painting performance ability of the experimental group and the control group [*F*_(1,58)_ = 14.128, *p* = 0.000] reached a significant level. It can be seen that the painting performance ability of the experimental group that received the children’s digital art ability training system with AI-assisted learning was significantly higher than that of the control group, thus, the research hypothesis is supported.

**TABLE 4:** Summary of the analysis of one-way covariance of “painting performance ability” of both groups of subjects.

Source	Type I sum of squares	*df*	Mean squares	*F*
Total score of drawing performance	34.12	1	16.92	14.128**

*p < 0.05 (significant); **p < 0.01 (highly significant); *** p < 0.001 (extremely significant).

## Conclusion

The results of this study show that the application of the children’s digital art ability training system with AI-assisted learning to the art learning activities in K-12 education can help improve students’ painting performance, and this system can improve the effectiveness of students’ art learning activities, as well as their performance in color recognition. The imagination (expressiveness, originality, and richness) and the quality of students’ works (relevance, feasibility, and color composition) have all been improved. In summary, the total score of the painting performance ability of the experimental group students who had received the children’s digital art ability training system with AI-assisted learning was significantly better than that of the control group students. According to the in-depth discussion on the content of the painting performance ability evaluation standards, the progress of students showed that this assisted learning system has good results in improving students’ painting performance ability.

## Data Availability Statement

The raw data supporting the conclusions of this article will be made available by the authors, without undue reservation.

## Ethics Statement

Written informed consent was obtained from the individual(s) for the publication of any identifiable images or data included in this article.

## Author Contributions

All authors listed have made a substantial, direct, and intellectual contribution to the work, and approved it for publication.

## Conflict of Interest

The authors declare that the research was conducted in the absence of any commercial or financial relationships that could be construed as a potential conflict of interest.

## Publisher’s Note

All claims expressed in this article are solely those of the authors and do not necessarily represent those of their affiliated organizations, or those of the publisher, the editors and the reviewers. Any product that may be evaluated in this article, or claim that may be made by its manufacturer, is not guaranteed or endorsed by the publisher.
